# Cyanoacrylate glue in breast surgery: the GLUBREAST Trial

**DOI:** 10.3389/fonc.2024.1473157

**Published:** 2025-01-20

**Authors:** Emanuela Esposito, Claudio Siani, Ivana Donzelli, Anna Crispo, Sergio Coluccia, Piergiacomo Di Gennaro, Assunta Luongo, Franca Avino, Alfredo Fucito, Ugo Marone, Maria Teresa Melucci, Ruggero Saponara, Raimondo di Giacomo

**Affiliations:** ^1^ Department of Breast and Thoracic Oncology, Istituto Nazionale Tumori, IRCCS-Fondazione G. Pascale, Naples, Italy; ^2^ Epidemiology and Biostatistics Unit, Istituto Nazionale Tumori - IRCCS "Fondazione G. Pascale", Naples, Italy

**Keywords:** cyanoacrylate glue, Glubran ^®^ 2, axillary seroma, axillary dissection, breast cancer

## Abstract

**Introduction:**

In 2018, the National Cancer Institute of Naples has launched the GLUBREAST Trial to verify the efficacy of cyanoacrylate sealing glue to prevent or reduce seroma after axillary dissection in breast surgery. The glue is a synthetic sealant (N-Butyl-2-CyanoAcrylate+Metacryloxisulfolane) biocompatible, CE approved for internal human uses and surgical procedures. The assumed mechanism of action in breast surgery is that the glue would create a seal coating in the operative field to occlude lymphatic leaks and limit seroma formation.

**Materials and methods:**

The trial included 180 patients scheduled for breast-conserving surgery or for radical modified mastectomy without reconstruction. Out of 180 patients, 91 were randomized to receive suction drain and sealant glue after axillary dissection (Experimental Arm), whereas 89 patients (Control Arm) received suction drain without glue.

**Statistics:**

A multivariable mixed effect model on presence of liquid drained and volume drained was calculated. Stratified models by visits were performed.

**Results:**

The trial ended in June 2022. Older age was associated with a higher volume of seroma drained per day (β 0.30; 95% CI: 0.00–0.60). A 5-U increase in body mass index was associated with higher daily drained seroma volume in patients who underwent breast-conserving surgery (β 5.0; 95% CI: 0.62–9.4), but not in patients who underwent mastectomy (β 2.5; 95% CI: −3.6–8.6). We did not find statistically significant differences in presence of liquid drained and volume drained among the study groups. An advantage for the Experimental Arm was observed from third and fourth to fifth outpatient visits without reaching a statistical significance (p=0.069 and p=0.072, respectively); so far, 5% of patients in the Experimental Group had clinical benefit from the glue.

**Conclusions:**

The vast majority of data in the literature come from case series, and surgeons need a higher level of evidence to drive surgical decision-making and choose proper devices to increase patient quality of life. The GLUBREAST randomized trial tested the efficacy of cyanoacrylate sealing glue to prevent postoperative seroma in breast surgery. Although only a small number of patients benefited from sealant application, we regret to say this trial has some limitation, i.e., the prolonged presence of suction drain. Further research is warranted to better clarify the benefit of cyanoacrylate glue in breast surgery.

## Introduction

Axillary seroma is the most frequent complication following breast surgery and axillary dissection. The formation of a seroma increases the number of outpatient visits and delays adjuvant therapies resulting in severe discomfort and stress for most of the patients. The incidence of seroma spans from 5% to 90% (1–4). Obesity, electrocautery, and big breast volume have been shown to be risk factors for seroma formation. Surgeons attempt to avoid seroma through many techniques: wound drainage, reduction of the dead space in the axillary cavity by closing carefully the different layers underneath the skin, use of various types of bipolar electrosurgical device, and external compression dressings (5).

Furthermore, several tools have been launched in the market with the same aim, such as the application of fibrin glue, somatostatin intramuscular injection, or oral cortison administration, without any noteworthy impact on the clinical practice (6). Cyanoacrylate glue was renowned for adhesive and hemostatic properties and shortly after was committed to reduce postoperative seroma along with lymphadenectomy. Glubran^®^ 2 is a synthetic cyanoacrylate sealant (N-Butyl-2-CyanoAcrylate+Metacryloxisulfolane) biocompatible, CE approved for internal human uses and surgical procedures (7). It is a Class III surgical medical device, widely used in open and laparoscopic surgery (liver; lung; colorectal) (8–10). It is also used as an embolizing agent in vascular surgery (9). Once applied, the glue polymerizes quickly and creates a film that conforms the target tissue. The polymerization process starts as the product spreads with wet environments, such as blood and tissues. The assumed mechanism of action is that the glue forms a seal coating in the operative field to occlude lymphatic leaks that limits seroma formation. Here, we present the GLUBREAST Trial (ISRCTN43919783), a prospective, single-center, randomized, controlled trial that evaluated the efficacy of Glubran^®^ 2 in preventing seroma among women undergoing either breast conserving surgery (BCS) and axillary dissection (AD) or radical modified mastectomy (MRM).

## Materials and methods

### Study design

We performed a single-center prospective randomized controlled trial at the Department of Breast and Thoracic Oncology of the “Istituto Nazionale Tumori, IRCCS-Fondazione G. Pascale” Naples, Italy. A total of 180 patients were enrolled in the GLUBREAST Trial.

#### Inclusion criteria

- female gender;
- 18 years of age or older;
- new diagnosis of invasive breast cancer confirmed by core needle biopsy;
- patients with palpable and positive axillary lymph nodes requiring axillary dissection;
- patients suitable for breast conserving surgery (BCS) or modified radical mastectomy (MRM) without reconstruction;
- ability of understanding and signing the informed consent.

#### Exclusion criteria

- male gender;
- patients who had been treated with neo-adjuvant chemotherapy;
- patients scheduled for breast reconstruction;
- patients previously treated with radiation therapy to the chest wall for lymphoma;
- inability to understand and sign the informed consent.

Neoadjuvant chemotherapy and breast reconstruction with either expanders or implants and matrices were exclusion criteria in order to create a sample as homogeneous as possible. The Internal Ethical Committee approved the study numbered 12/18 in 2018 (CE Approval document is available and can be sent if requested).

#### Randomization

After signing the informed consent, patients were randomly assigned to Experimental Arm or to Control Arm through an allocation program accessible at the following link: https://glubreast.ibisinfo.online/Public/homepage.php.

**Figure 1:**
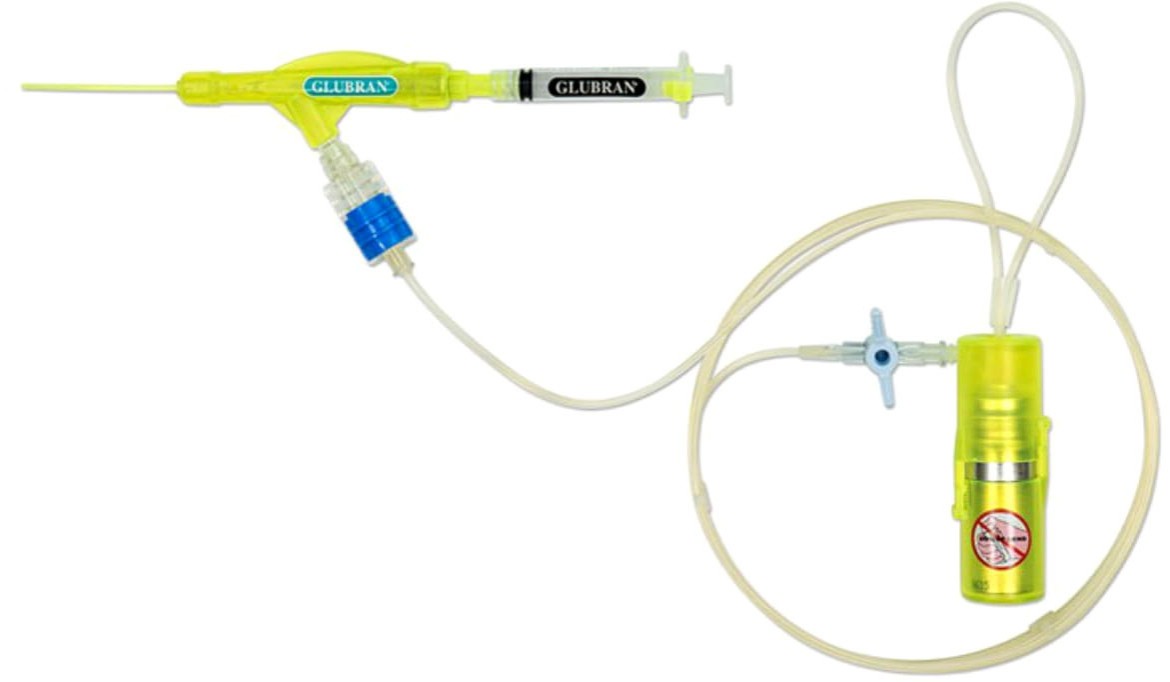
Spray device used in open surgery to nebulize cyanoacrylate glue on the surgical field.

**Figure 2:**
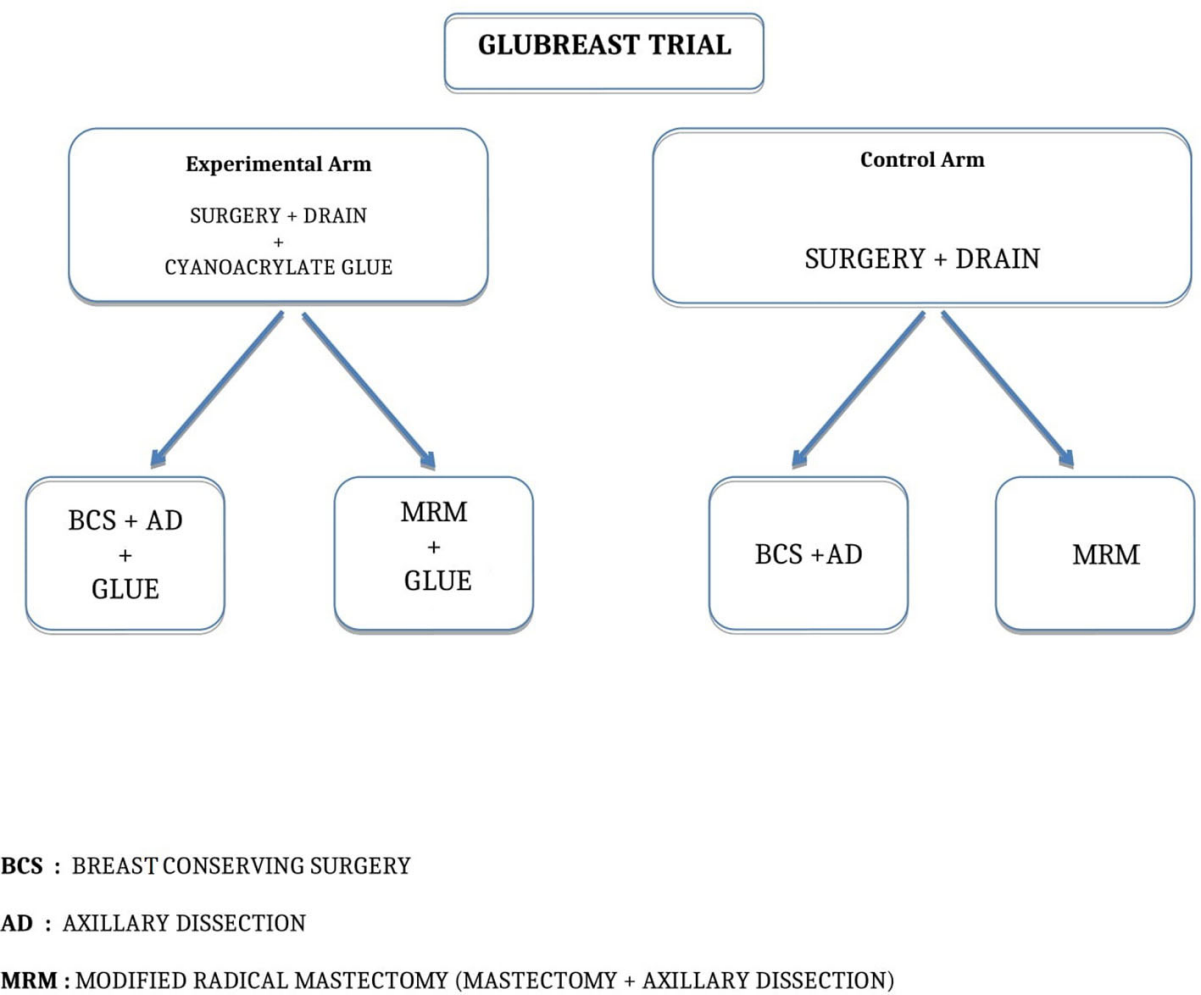
GLUBREAST Trial flowchart.

After completing breast resection and axillary dissection for levels I and II, stopping at the medial border of pectoralis minor and including the third level only if lymph nodes were grossly involved, surgeons applied one milliliter (ml) of cyanoacrylate glue as a spray by a disposable nebulizer device with a gas autonomous propulsion system in the axillary cavity of patients randomized in the Experimental Arm (**Figure 1**). Once the glue had crystallized on the empty axillary cavity, a suction drain was placed within the cavity and the wound was closed in three layers. Surgeons did not apply the glue in the Control Arm, so far after completing breast resection and axillary dissection, placed a suction drain, and closed the wound in three layers. **Figure 2** shows the trial flowchart.

Patients were followed up every week until seroma was completely absorbed and then at 3 months after surgery. In order to measure the actual volume of seroma within the cavity, the suction drain was removed on day 15 after surgery for all patients in both arms. In case of persistent seroma after drain removal, the remaining serum was collected by fine needle aspiration and volumes aspirated were reported in the eCRF along with the number of visits and adverse events forms were filled out. If drain output was less than 50 ml before day 15, the patient would have her drain removed and excluded from the study. However, in our daily clinical practice, we tend to use suction drain for 15 days or more since the majority of patients have BMI >30 kg/m^2^ and it is absolutely rare we remove drain before day 15 after axillary clearance.

### Primary endpoints

- Efficacy of cyanoacrylate glue to prevent postoperative seroma in breast surgery in terms of total volume drained

### Secondary endpoints

- Reduction of seroma after drain removal by measuring the volume of seroma through fine needle aspiration
- Correlation of seroma with body mass index (BMI)
- Correlation of seroma with type of breast surgery
- Number of adverse events after glue application
- Rate of infection; hospital readmission and reoperation after glue application

### Sample size calculation

The sample size was calculated based on the primary endpoint of the study, which was the total volume of drained seroma (ml) during the first 15 days following surgery. The significance level (alpha) was set at 5%. Assuming two groups (the Experimental Arm group and the Control Arm, with and without application of Glubran^®^ 2, respectively) each consisting of 100 patients, this design provides at least 80% power to detect a difference of 0.4 on the primary endpoint, corresponding to a small-to-medium effect size, using a two-tailed t-test for independent samples. The final sample size was increased to 110 patients per group to minimize the impact of potential dropouts during follow-up.

### Statistical analysis

Differences between study arms were evaluated through Pearson’s chi-squared test or Fisher’s exact test (if an exact test was needed) for categorical variables and Mann–Whitney test for continuous variables. Adverse events and presence of liquid drained were shown as occurrence in at least one visit per patient. Volume drained was shown as volume (ml/day) mean in all visits. To investigate the independent contribution of predictors on presence/absence of liquid (expressed as risk of seroma formation) and volume drained (expressed as daily drained seroma volume), a multivariable mixed effect model for repeated measures was performed including the following covariates: age, tumor size, body mass index (BMI), time to visit, kind of surgery, surgical complications; significance of each coefficient was evaluated through t-statistics using Satterthwaite’s method. Regression stratified by kind of surgery was also performed. Statistical significance was set at p < 0.05. A Kaplan–Meier with a log-rank test was performed to investigate the difference in time to last drainage among the study groups. Analyses were performed using R software 4.2.3.

## Results

### Univariable analysis

This prospective, randomized clinical trial enrolled 180 patients with a newly diagnosed invasive breast cancer. Among these, 91 patients were randomized to the Experimental Arm (MRM or BCS plus axillary dissection along with application of Glubran^®^ 2) and 89 patients to the Control Arm (MRM or BCS plus axillary dissection alone, without application of Glubran^®^ 2). **Table 1** summarizes the baseline characteristics of participants. We did not find statistically significant differences between the groups at the enrollment. Mean age of participants was 64 ± 13 years in both groups. According to BMI categories, 66 patients (73%) in the Experimental Arm and 59 patients (66%) in the Control Arm were overweight or obese (BMI >25 kg/m^2^) with BMI means of 28.5 kg/m^2^ and 28.4 kg/m^2^, respectively. Regarding tumor characteristics, the mean tumor size was 9 ± 11 mm ranging from 1 mm to 50 mm in both groups. The median number of nodes removed was 13 (range 7–28). The median number of positive nodes removed was 3 (range 1–14). Only 7.5% of patients had level III axillary clearance. 92.5% of patients underwent axillary clearance of levels I and II. The average length of recovery from surgery was 32 ± 9 days in the Experimental Arm and 33 ± 9 in the Control Arm. There were 10 patients (11%) in the Experimental Arm and five patients (5.6%) in the Control Arm who had previous surgery for breast cancer in other institution and completed with axillary surgery in our institution. A total of 56 patients (31.1%) underwent MRM. There were 29 out of 56 who were allocated in the Experimental Arm (32%), whereas 27 out of 56 patients (30%) were allocated in the Control Arm. A total of 124 patients (68.9%) underwent BCS. There were 62 out of 124 (68%) who were allocated in the Experimental Arm, whereas 62 out of 124 (70%) were allocated in the Control Arm. Lastly, 29 patients in the Experimental Arm and 31 patients in the Control Arm received either anticoagulant or antiplatelet therapy (**Table 1**).

**Table 1:** main baseline characteristics by treatment arm.

Variable	Control Arm N = 89	Experimental Arm GLUBRAN2 N = 91	p-value^1^
Age			>0.9
*Mean (SD)*	64 (13)	64 (13)	
*Range*	36, 87	31, 92	
BMI			0.7
*Mean (SD)*	28.4 (5.8)	28.5 (5.2)	
*Range*	19.0, 50.2	17.8, 44.4	
BMI Classification			0.4
*Overweight-Obesity (>25)*	59 (66%)	66 (73%)	
*Until normal weight (<25)*	30 (34%)	25 (27%)	
Tumor Size			0.9
*Mean (SD)*	9 (11)	9 (11)	
*Range*	1, 50	1, 50	
*Unknown*	4	0	
Type of surgery			0.8
*Modified Radical Mastectomy*	27 (30%)	29 (32%)	
*Breast Conserving Surgery*	62 (70%)	62 (68%)	
Patient RecoveryDuration			0.2
*Mean (SD)*	33 (9)	32 (9)	
*Range*	20, 62	20, 66	
*Unknown*	9	2	
Previous breastintervention			0.2
*No*	84 (94%)	81 (89%)	
*Yes*	5 (5.6%)	10 (11%)	
Menopausal status			0.5
*Post*	70 (79%)	75 (82%)	
*Pre*	19 (21%)	16 (18%)	
Smoker			0.2
*No*	78 (91%)	74 (84%)	
*Yes*	8 (9.3%)	14 (16%)	
*Unknown*	3	3	
Alcohol and/or psychotropic substances user			0.7
*No*	82 (98%)	79 (96%)	
*Yes*	2 (2.4%)	3 (3.7%)	
*Unknown*	5	9	
Arthropathy			0.8
*No*	49 (56%)	52 (57%)	
*Yes*	39 (44%)	39 (43%)	
*Unknown*	1	0	
Diabetes			0.5
*No*	76 (85%)	74 (81%)	
*Yes*	13 (15%)	17 (19%)	
Cardiopathy			0.9
*No*	64 (76%)	67 (77%)	
*Yes*	20 (24%)	20 (23%)	
*Unknown*	5	4	
HCV+			0.2
*No*	84 (95%)	82 (90%)	
*Yes*	4 (4.5%)	9 (9.9%)	
*Unknown*	1	0	
HBsAg+			>0.9
*No*	88 (99%)	90 (99%)	
*Yes*	1 (1.1%)	1 (1.1%)	
Anticoagulant therapy			0.9
*No*	76 (88%)	81 (89%)	
*Yes*	10 (12%)	10 (11%)	
*Unknown*	3	0	
ASA			0.6
*No*	66 (76%)	72 (79%)	
*Yes*	21 (24%)	19 (21%)	
*Unknown*	2	0	

^1^Wilcoxon rank sum test; Pearson's Chi-squared test; Fisher's exact test.

A small percentage of patients developed symptoms: in particular, fever was observed in two patients (2.2%) in both study groups. Seven patients (7.7%) in the Experimental Arm and six patients (6.7%) in the Control Arm experienced breast and axillary pain. Infections were detected in three patients each group (3.3% and 3.4%, respectively). Regarding pharmacological treatment, only four patients (4.4%) in the Experimental Arm and three patients (3.4%) in the Control Arm received postoperative antibiotic therapy. Similarly, only three patients (3.3%) in the Experimental Arm and one patient (1.1%) in the Control Arm developed skin wound dehiscence; however, we did not find statistically significant differences among the groups in symptoms and side effects. The mean daily drained seroma volume (ml/day) was slightly higher (p=0.035) in the Experimental Arm compared with the Control Arm (53 ± 43 ml/day vs. 47 ± 42 ml/day). **Table 2** shows the number and percentage of participants who experienced symptoms and side effects.

**Table 2:** Side effects by treatment arm.

Arm			
Variable	Control Arm N = 89^1^	Experimental Arm GLUBRAN2 N = 91^1^	p-value^2^
Fever			>0.9
*No*	87 (98%)	89 (98%)	
*Yes*	2 (2.2%)	2 (2.2%)	
Pain			0.8
*No*	83 (93%)	84 (92%)	
*Yes*	6 (6.7%)	7 (7.7%)	
Infection			>0.9
*No*	86 (97%)	88 (97%)	
*Yes*	3 (3.4%)	3 (3.3%)	
Antibiotic treatment			>0.9
*No*	86 (97%)	87 (96%)	
*Yes*	3 (3.4%)	4 (4.4%)	
Skin wound dehiscence			0.6
*No*	88 (99%)	88 (97%)	
*Yes*	1 (1.1%)	3 (3.3%)	
Liquid drained			0.5
*No*	1 (1.1%)	0 (0%)	
*Yes*	88 (99%)	91 (100%)	
Volume drained mean (ml/day)			0.034
*Mean (SD)*	47 (42)	53 (43)	
*Median (IQR)*	40 (12, 66)	50 (19, 76)	

^1^n (%).

^2^Fisher's exact test; Pearson's Chi-squared test; Wilcoxon rank sum test.

### Multivariable analysis

In multivariable analysis, we first investigated the linear association between patients’ characteristics and the risk of seroma formation (**Table 3**, Model 1). In this first model, age and tumor size were not associated with an increased risk of seroma formation. Similarly, we did not find differences between the study groups (OR 0.87; 95% CI: 0.45–1.68). A higher BMI evaluated as a 5-U increment was not significantly associated with an increased risk of seroma formation (OR 1.07; 95% CI: 0.78–1.47). As we expected, time of outpatient visits (in days) was significantly and inversely associated with risk of postoperative seroma formation (OR 0.84; 95% CI: 0.80–0.88; p<0.001).

**Table 3:** Multivariable models results for risk of seroma formation (Model 1) and volume of drained seroma (ml) (Model 2).

Characteristic	Model 1 Liquid Presence			Model 2 Volume Drained		
	OR^1^	95% CI	p-value	Beta^2^	95% CI	p-value
***PAT_AGEYEAR***	0.99	0.96, 1.01	0.332	0.30	0.00, 0.60	**0.050**
***TUMOR_SIZE***	1.01	0.98, 1.04	0.712	-0.12	-0.46, 0.22	0.479
***RANDOMIZATION_ARM***			0.668			0.102
Control Arm	—	—		—	—	
Experimental Arm GLUBRAN2	0.87	0.45, 1.68		6.1	-1.3, 13	
***BMI***	1.07	0.78, 1.47	0.676	4.2	0.76, 7.6	**0.016**
***VISIT_TIME***	0.84	0.80, 0.88	**<0.001**	-2.1	-2.4, -1.9	**<0.001**
***TYPE OF_SURGERY***			0.365			0.172
MRM	—	—		—	—	
Breast Conserving surgery	0.70	0.33, 1.51		5.9	-2.6, 14	
***INFECTION***			0.962			0.092
No	—	—		—	—	
Yes	1.05	0.15, 7.19		18	-3.2, 40	

^1^Odds Ratio (OR) and 95% Confidence Interval (95%CI) were estimated through a regression model.

^2^Beta and 95% Confidence Interval (95%CI) were estimated through a multivariable mixed effect model. Bold values indicate statistical significant results.

Subsequently, we investigated the linear association between patients’ characteristics and the mean daily drained seroma volume (**Table 3**, Model 2). In this second model, age was associated with a higher volume of seroma drained per day (β 0.30; 95% CI: 0.00–0.60). As previously observed, we did not find statistically significant differences among the study groups (β 6.10; 95% CI: −1.30–0.60). Higher BMI was associated with daily drained seroma volume: in particular, each 5-U increase in BMI was associated with a higher daily drained seroma volume (β: 4.20; 95% CI; 0.76–7.60). Moreover, time of outpatient visits was significantly associated with a reduction in daily drained seroma volume (β −2.10; 95% CI: −2.40, −1.90; P<0.001).

When we investigated the associations between daily drained seroma volume and patients’ characteristics according to breast cancer surgery (**Table 4**), we confirmed the association between BMI and higher drained seroma volume per day. In particular, each 5-U increase in BMI was associated with higher daily drained seroma volume in patients underwent BCS (β 5.0; 95% CI: 0.62–9.4), but not in patients who underwent MRM (β 2.5; 95% CI: −3.6–8.6) with a p value of 0.024. In the multivariable model results for volume of drained seroma (ml) by type of surgery, time of outpatient visits was also significantly associated with a reduction in daily drained seroma volume both in patients underwent MRM and BCS (β −2.0; 95% CI: −2.5, −1.6 and β −2.2; 95% CI; −2.5, −1.9, respectively, P<0.001). Patients with a larger tumor size (>4 cm) who underwent BCS in the Experimental Arm were found to have higher daily drained seroma compared with the Control Arm (β 21.0; 95% CI; 6.6–35.0).

**Table 4:** Multivariable models results for volume of drained seroma (ml) by type of surgery.

Characteristic	Volume Drained in MRM			Volume Drained in BCS		
	Beta^1^	95% CI	p-value	Beta^1^	95% CI	p-value
**PAT_AGEYEAR**	0.25	-0.33, 0.83	0.383	0.29	-0.08, 0.67	0.118
**TUMOR_SIZE**	-0.22	-0.87, 0.43	0.493	-0.06	-0.48, 0.35	0.764
**RANDOMIZATION_ARM**			0.720			0.098
Control Arm	—	—		—	—	
Experimental Arm GLUBRAN2	2.6	-12, 17		7.4	-1.5, 16	
**BMI**	2.5	-3.6, 8.6	0.414	5.0	0.62, 9.4	**0.024**
**VISIT_TIME**	-2.0	-2.5, -1.6	**<0.001**	-2.2	-2.5, -1.9	**<0.001**
**INFECTION**			0.453			0.111
No	—	—		—	—	
Yes	14	-23, 51		23	-5.6, 52	

^1^Beta and 95% Confidence Interval (95%CI) were estimated through a multivariable mixed effect model.

Bold values indicate statistical significant results.

Finally, we did not find statistically significant differences in time to drain removal among the study groups, but this was due to the timing of drain removal set at day 15 for both Arms in the trial design. This might represent a limitation of the study.

However, none of the patient presented with less than 50 ml within the suction drain before day 15. If this situation would have happened, drain should have been removed and the patient excluded from the trial. However, in our daily clinical practice, we tend to leave suction drain in site for roughly 15 days or more since the majority of patients have BMI >30 kg/m^2^ and it is absolutely rare we remove drain before day 15 after axillary clearance (**Figure 2**).

**Figure 3:**
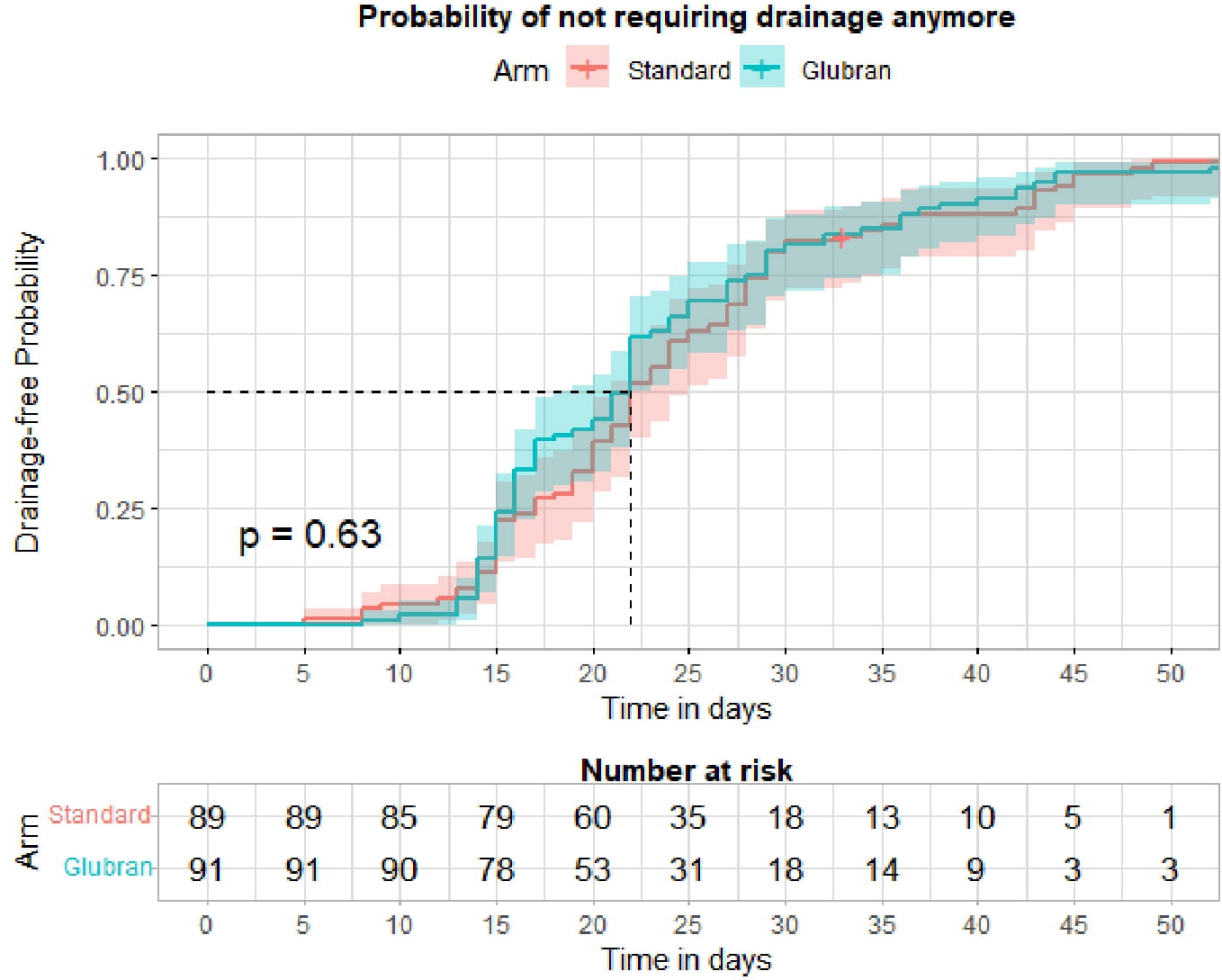
Kaplan-Meier curve and Log-rank test for time to drain removal by study arm.

**Figure 4:**
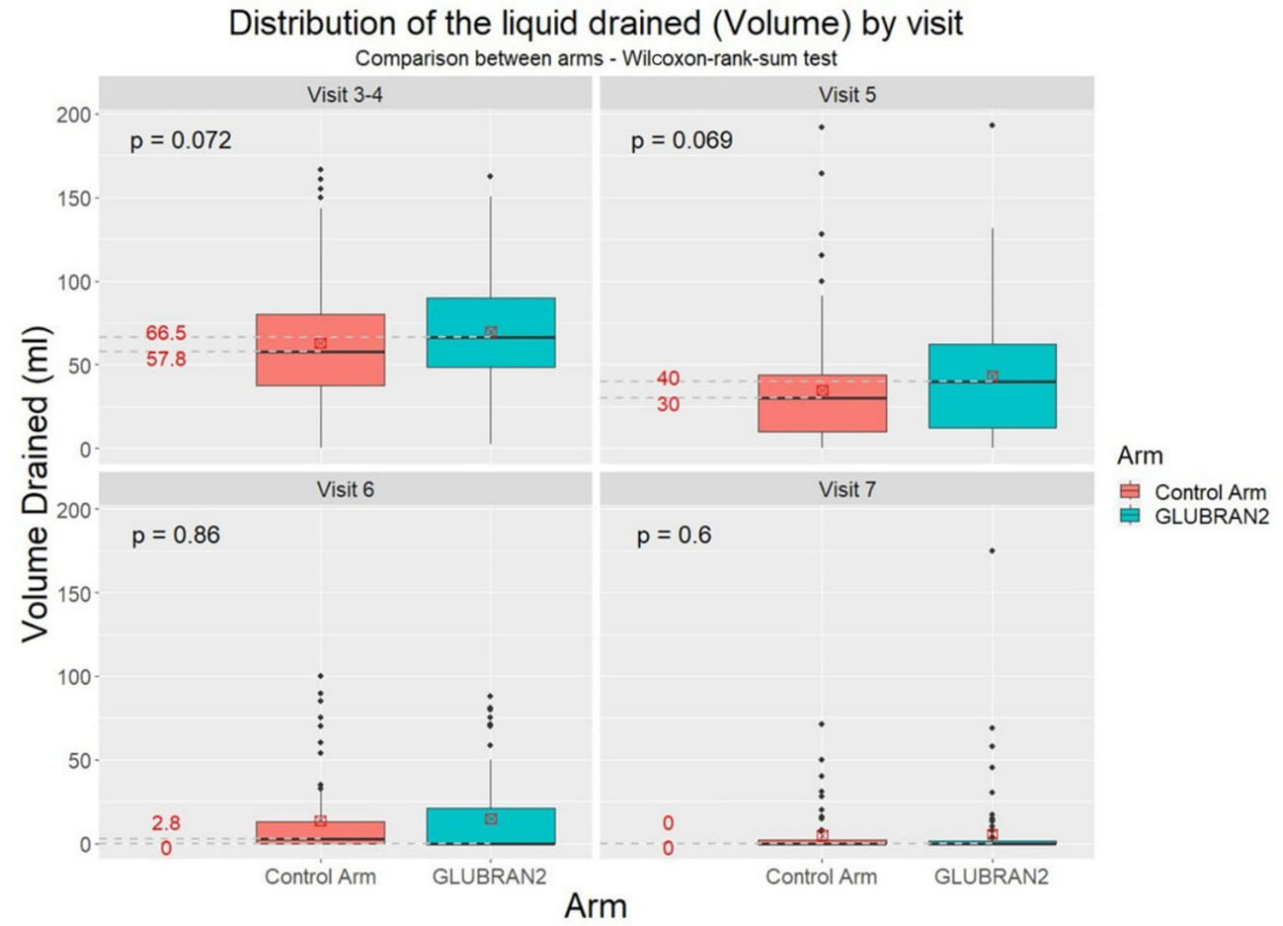
Distribution of total seroma volume drained in the study groups at the 3^th^–4^th^, 5^th^, 6^th^, and 7^th^ follow-up visits.

We further investigated the distribution of total seroma volume drained in the study groups at the third, fourth, fifth, sixth, and seventh follow-up outpatient visits (**Figure 3**). An advantage for the Experimental Arm was observed from the third and fourth to fifth follow-up visits without reaching a statistical significance (p=0.069 and p=0.072, respectively); so far, 5% of patients in the Experimental Group had clinical benefit from the glue, as shown in **Figure 3**. The total seroma volume drained was slightly higher in the Experimental Arm compared with the Control Arm at the third to fourth (66.5 ml vs. 57.8 ml, respectively) and fifth follow-up visits (40.0 ml vs. 30.0 ml, respectively), but these results did not reach statistical significance. However, we found that total seroma volume drained was lower in the Experimental Arm compared with the Control Arm at the sixth outpatient visit (0 ml vs. 2.8 ml, respectively), although this result was not statistically significant (**Figure 4**).

## Discussion

In the current trial, the use of Glubran^®^ 2 in patients undergoing breast cancer surgery and axillary dissection was not independently associated with seroma formation or seroma volume drained per day. Both the study arms were comparable in terms of age, BMI, comorbidities, and other clinical characteristics except for drained seroma volume, which was slightly higher in the Experimental Arm compared with the Control Arm; thus, the trial failed to meet the primary endpoint.

To the best of our knowledge, few randomized and controlled clinical trials tested the efficacy of Glubran^®^ 2 on seroma formation in breast cancer surgery.

De Luca et al. (11) did not find any evidence in the use of surgical glues to reduce the formation of seroma following axillary dissection in breast cancer patients. Nevertheless, they reported that the use of cyanoacrylate glue in association with closed suction axillary drain seems to contribute to the reduction in days of axillary drain permanence (7 days vs. 14 days) and of postoperative infections, which are factors delaying the schedule of any adjuvant oncological therapies (11).

Clement at al (12). did not find any benefit with the use of Glubran^®^ 2 in mastectomy and axillary surgery in reducing the risk of seroma formation but reported an increase in seroma formation and postoperative wound infection among elderly and obese patients with the use of Glubran^®^ 2. In our trial, Glubran^®^ 2 did not increase infection rates in the Experimental Arm.

In our experience, the use of Glubran^®^ 2 did not result in an increase of symptoms and/or side effects in the Experimental Arm confirming its safety.

In the study of Al-Masri et al. (13), the use of Glubran^®^ 2 did not influence seroma formation in patients undergoing axillary dissection, but it was associated with earlier drain removal. Despite the abovementioned results, according to Vasileiadou et al. (14), the use of Glubran^®^ 2 significantly decreases seroma production, drainage amount, and time to drain removal in patients undergoing to breast cancer surgery and lymph node dissection. Furthermore, the authors identified age, tumor size, and BMI as the main contributors associated with the seroma production (14).

The latter findings are consistent with our study results. In particular, we found that age was associated with higher daily drained seroma volume. Larger tumor size (>4 cm) was associated with higher daily drained seroma volume in patients who underwent BCS within the Experimental Arm. Moreover, in the current trial, we found a statistically significant association between BMI and higher seroma volume. In particular, each 5-U increase in BMI was associated with higher daily drained seroma volume in patients who underwent BCS, but not in patients who underwent MRM. Similar findings were also found in the study of Al-Masri et al. (13) in which higher BMI (≥30 kg/m^2^) was identified as an independent predictor of a higher daily drained seroma volume, both in the Experimental group and in the Control group. A condition of overweight and obesity, according to a BMI greater than 25 kg/m^2^, has long been associated with adverse medical events and surgical complications, in the seroma formation. In the study of Sforza et al. (15), 50% of obese patients developed seroma compared with 1.89% of patients with a lower BMI. Consistent with that in the study by Chen et al. (16), a condition of obesity was associated with a 10-fold increased risk of seroma formation. These results not only confirmed obesity’s role as a major predictor of negative surgical outcomes but also highlighted its association with seroma.

Finally, we found that time of outpatient visits was independently associated with a reduction in daily drained seroma volume, overall and according to breast cancer surgery, in patients who underwent both BCS and MRM. Moreover, we found that at third and fourth to fifth outpatient visits, the Experimental Arm presented with a slightly higher volume of seroma not statistically significant, whereas at the sixth visit, which coincided with the third week after surgery, the Experimental Arm showed a lower seroma volume drained compared with the Control Arm. This finding may suggest different behaviors among the two arms at face with the wound healing that needs to be further investigated. Considering that this trial failed to reach its primary endpoint, some limitations that might have affected results need to be stated. Firstly, we have to remember our sample consisting mostly of women who were overweight and obese with an average BMI of 28.5 kg/m^2^ ranging from 17.8 kg/m^2^ to 44.4 kg/m^2^ in the Experimental Arm. This condition may have resulted in higher seroma production and may have reduced the efficacy of the cyanoacrylate glue.

Secondly, during trial design, drain removal was set at day 15 after surgery in both the study arms, to measure seroma volume accurately in ml. We tend to keep drain till day 15 after axillary clearance in the daily clinical practice as the majority of patients, which are overweight and obese have daily output higher then 50/ml per day till day 15 or more. As a consequence of this, the presumed efficacy of Glubran^®^ 2 on early drain removal has not been evaluated, and this is to be considered a limitation of the study.

Furthermore, the lack of statistically significant differences between the two arms could be associated with the suboptimal sample size of the study.

On the other hand, the present trial has several strengths. This was a prospective, randomized, and controlled clinical trial, and data collection was accurate and detailed. Moreover, our study offers an accurate definition of the timing of outpatient visits and information about the safety of the glue.

Taken together, these results emphasize the need of a further clinical, randomized, and multicenter trial with a larger sample size and earlier surgical drain removal or even drainless procedure to investigate the efficacy of Glubran^®^ 2 in the reduction of seroma after breast cancer surgery in order to improve patient’s quality of life.

## Data Availability

The raw data supporting the conclusions of this article will be made available by the authors, without undue reservation.
